# Conductive impairment in newborn who failed the newborn hearing screening

**DOI:** 10.1590/S1808-86942010000300013

**Published:** 2015-10-20

**Authors:** Priscila Karla Santana Pereira, Marisa Frasson de Azevedo, José Ricardo Testa

**Affiliations:** 1 Master's degree, speech therapist; 2 Speech therapist, doctoral degree, adjunct professor IV at the Sao Paulo Federal University/UNIFESP/Paulista Medical School/EPM; 3 Otorhinolaryngologist, doctoral degree, adjunct professor of otorhinolaryngology at the Sao Paulo Federal University/UNIFESP/Paulista Medical School/EPM

**Keywords:** child, neonatal screening, hearing

## Abstract

In newborn hearing screening little importance is attributed to changes in the middle ear. Children with secretory otitis in the neonatal period are at risk for developing otitis media in the first year of life.

**Aim:** To determine if children who failed the hearing screening because of conductive hearing loss have more episodes of conductive hearing impairment during their first years of life.

**Materials and Methods:** The study group comprised 62 children who failed the screening for conductive impairment. The control was made up of 221 who passed. Both had audiologic and otolaryngological assistance and were compared regarding the occurrence of conductive disorder. Were used the Fisher's Exact test for statistical analysis and logistic regression models. The study was prospective and retrospective.

**Results:** Children who failed the screening by conductive disorder had more episodes of otitis media during the first year of life than those who did not fail, with statistically significant difference.

**Conclusion:** Infants who failed the screening in the first month of life for conductive alteration are more likely to experience otitis in the first year of life. The high incidence of otitis indicates the need for joint action with otolaryngologist for diagnosis of such changes.

## INTRODUCTION

Anatomical and physiological integrity of the central and peripheral auditory system is a prerequisite for normal language acquisition and development (Azevedo, 1996).^1^

Therefore, the impact of undetected hearing loss on the development of language and socialization of children has given rise to neonatal screening programs.

Northern and Downs (1991)^2^ underlined the importance of neonatal hearing screening for identifying congenital hearing loss and middle ear changes; these alterations in especially the first year of life affect speech and language development in children.

Intermittent alterations due to conductive hearing loss because of recurring otitis may affect the ability to process sound stimuli; in such cases, acoustic parameters vary according to temporary or period hearing loss (Bamford, Saunders, 1997).^3^ Thus, children with recurrent and/or persistent otitis media within the first two years of life are at a risk for hearing loss.

Secretory otitis media or otitis media with effusion is the most frequent cause of conductive hearing loss in children in developed countries. It may be defined as the presence of fluid in the middle ear with no signs or symptoms of acute infection that occurs in the first years of life. It may be accompanied by mild to moderate episodic, variable, conductive hearing loss never over 50 dB (Saes et al., 2005).^4^

Secretory otitis media is rare in the first 6 months of life. Its incidence increases markedly from 6 to 12 months of age, reaching about 13 to 15% at age 12 months. The peak frequency occurs from age 2 to 4 years; at this age, 20% of all children experience secretory otitis media (Fiellau-Nikolajsen, 1990).^5^

Little importance has been given to middle ear alterations – which are transitory – in neonatal hearing screening programs. Doyle et al. (2004)^6^ has reported that failures in auditory screening as tested by transient-evoked otoacoustic emissions may also be attributed to middle ear changes (secretory otitis), and that children with secretory otitis media during the neonatal period are at an increased risk of developing chronic otitis media within the first year of life.

Sensory deprivation due to secretory otitis media may affect speech perception and understanding, especially in noisy ambiences; Language development may also be affected. This is further compounded by the number and duration of episodes of this disease.

Fluctuating hearing loss due to middle ear diseases in the first years of life may result in altered auditory processing, which affects learning.

Thus, prevention, detection, and especially monitoring for at least a year is essential for the newborn that failed in conductive hearing screening tests. Middle ear alterations (otitis), which peak from ages 6 to 12 years, may persist; this age range is important because this is when the auditory system and language acquisition take place.

Therefore, the purpose of this study was to verify whether neonates that failed the conductive test hearing screening are more likely to present middle ear alterations during the first year of life, compared to those that passed hearing screening.

## MATERIAL AND METHOD

The institutional review board analyzed and approved this study on 21 December 2007 (protocol no. 1561/07).

Parents and/or caretakers that agreed to have their children participate in this study were duly informed and signed a free informed consent form, as required by the ethics of research in human beings.

Children that presented every six months for hearing monitoring were included; these children underwent at least three assessments of hearing (on the 1st, 6th and 12th months of life). Children with neurological conditions, malformations, or sensorineural hearing loss were excluded from this study. The study population was allocated to two groups: a control group consisting of children that passed neonatal hearing screening, and a study group consisting of children that failed neonatal hearing screening, which was confirmed by tympanometry and an otorhinolaryn-gological evaluation.

This was a retrospective and prospective study. For the retrospective study, the medical files of 217 preterm children born from 2003 to 2006 were assessed; these children were seen in the multidisciplinary monitoring program for high risk neonates. The prospective study consisted of following-up 66 children born in 2007, of which 31 were term births and 35 were born preterm.

Both groups were compared to test the hypothesis that children in the study group had a higher rate of conductive hearing loss because of secretory otitis media within the fits year of life, compared to controls. The frequency of conductive hearing loss in children that failed in the hearing screening due to conductive loss was analyzed during the 12-month monitoring period.

The following tests were applied for testing hearing: transient otoacoustic evoked emissions (TOAE), immittance testing at 226Hz, behavioral observation, investigation of the cochleo-palpebral reflex, visual reinforcement audiometry, and an otorhinolaryngological evaluation.

An ILO 292 – Otoacoustic Emissions Analyzer (Otodynamics ANALISER Ltd, version 4.2) coupled to a conventional computer was used for TOAE testing. TOAE were considered as present when the signal-to-noise ratio was equal to or higher than 3 dB at 1,500 Hz and equal to or higher than 6 dB at 2,000 Hz, 3,000 Hz and 4,000 Hz, and when general reproducibility was equal to or higher than 50% with sound stability equal to or higher than 70%, as proposed by Finitzo et al. (1998)7 and recommended by Azevedo (2003).^8^

An Interacoustics AZ7 manual impedance audiometer (226 Hz probe) was used for immittance testing. A type A tympanometric curve was considered as normal (Jerger 1970);9 type B and C curves were considered as altered (Jerger 1970).^9^

The behavioral assessment consisted of a survey of attention responses, source searching and location of music instrument sounds from 50 to 90 dBSPL, and investigation of the cochleo-palpebral reflex with agogô bells (100 dBNPS); Azevedo's (1995) age adaptation criterion was applied.^10^

An Interacoustics PA 5 pediatric audiometer was used for visual reinforcement audiometry in children aged from 6 to 12 months at 500 Hz, 1,000 Hz, 2,000 Hz and 4,000 Hz in free field. The stimulus was delivered 20 cm from the right and left ear lobes at decreasing intensities; a red light stimulus was delivered when the child moved the head towards the source of sound. The normal range in the free field evaluation was 40-60 dBHL in children aged from 6 to 9 months, and 20-40 dBHL in children aged from 9 to 13 months (Azevedo, 2004).^11^

The otorhinolaryngological evaluation consisted of otoscopy: an otorhinolaryngologist examined the newborn with an otoscopy to assess the status of the outer ear canal and the tympanic membrane. For our purposes, the tympanic membrane was classified as normal or altered (retracted, hyperemic, opaque, perforated, and bulging). The physician that carried out these exams has over 15 year's professional experience with neonates. Failure due to conductive hearing loss in screening at birth was defined when TOAE and the cochleo-palpebral reflex were absent, when there was a type B tympanometric curve, when otoscopy was consistent with secretory otitis media, and when post-therapeutic/surgical exams were normal. Conductive hearing loss was defined as present when elevated pure tones for the age evoked minimal responses, TOAE and the cochleo-palpebral reflex were absent, and tympanic curves and otoscopy were altered.

The logistic regression model and Fisher's exact test were applied to verify whether there were statistically significant differences on the occurrence of conductive hearing loss between the groups. Differences were considered as statistically significant differences when the p-value was below (5%). Significant values are marked with an asterisk (*).

## RESULTS

### Prospective Study

The prospective study consisted of monitoring 66 children who were born in 2007; 31 were female and 35 were male; 31 were term births and 35 were pre-term births.

Table 1 shows the occurrence of conductive hearing loss within the first year of life in the control group.

**Table 1:** Occurrence of conductive hearing loss in the study groups

Group	Number of children	Number of children with condutive loss	%	P-Value	Odds Ratio
Control	38	9	23,7	0,0097*	4,29
Study group	28		16	57,1	
Total	66		25	37,9	

Analysis of the occurrence of conductive hearing loss in each group revealed that there was a statistically significant difference (p=0.0097*). The chance of a newborn in the control group to not present conductive hearing loss within the first year of life was 4.29 times higher than the chance of a newborn in the study group to not present conductive hearing loss (Table 1).

Table 2 shows the number of episodes of conductive hearing loss due to otitis. The peak age in which otitis occurred in the control group was 12 months, while in the study group this age was 6 months.

**Table 2:** Occurrence of otitis

Group	Number of episodes of otite in the second assessment (6 months)	Number of episodes of otite in the third assessment (12 months)	Total no. of episodes of otitis
Control group	6 (46,1%)	7 (53,8%)	13 (100%)
Study group	11 (55%)	9 (45%)	20 (100%)
Total	17	16	33

Table 3 shows the number of children with one or two episodes of conductive hearing loss during the study. Most of the children had at least one episode of otitis in 12 months of monitoring newborn babies after hearing screening.

**Table 3:** Number of children according to episodes of conductive hearing loss in both groups

Group	Children with one episode of otitis	Children with two episodes of otitis	Children that did not present otitis	Total
Control group	5 (13,1%)	4 (10,5%)	29 (76,3%)	38 (100%)
Study group	12 (42,8%)	4 (14,2%)	16 (57,1%)	28 (100%)
Total	17	8	45	66

Tables 4 and 5 show the occurrence of conductive hearing loss according to sex. The highest occurrence was in males in both groups, but this difference was not statistically significant.

**Table 4:** Occurrence of conductive hearing loss according to gender in the control group

			Number of children with conductive loss	
Sex		n	%	P-Value
Female		4	44,4	0,6329
Male		5	55,5	
Total		9	100	

Key: n = number

**Table 5:** Occurrence of conductive hearing loss according to gender in the control group

			Number of children with conductive loss	
Sex		n	%	P-Value
Female		6	37,5	1,0000
Male		10	62,5	
Total		16	100	

Key: n=number

### Retrospective Study

The retrospective study consisted of analyzing the medical files of 217 children of preterm births from 2003 to 2006; there were 115 male and 102 female subjects, all included in the multidisciplinary monitoring program for high risk neonates. The study group comprised children that failed the hearing screening assessment because of conductive hearing loss; the control group consisted of children that did not fail in hearing screening.

Table 6 shows the occurrence of conductive hearing loss within the first year of life in the control group. Analysis of the occurrence of conductive hearing loss in each group revealed that there was a statistically significant difference (p=0.0186*). The chance of a control group newborn not presenting conductive hearing loss was 2.9 times higher compared to the chance of a study group newborn.

**Table 6:** Occurrence of conductive hearing loss in both groups

Group	Number of children in the study	Number of children with conductive loss	%	P-Value	Odds Ratio
Control group	183	23	12,6	0,0186*	2,9
Study group	34	10	29,4		
Total	217	33	15,2		

Table 7 shows the number of episodes of conductive hearing loss because of otitis in each assessment. The age of peak occurrence was 6 months in both groups.

**Table 7:** Occurrence of otitis

Group	Number of episodes of otitis in the second assessment (6 months)	Number of episodes of otitis in the third assessment (12 months)	Total no. of episodes of otitis
Control group	10 (76,9%)	14 (23,1%)	24 (100%)
Study group	7 (53,8%)	6 (46,1%)	13 (100%)
Total	17	20	37

Table 8 shows the number of children with one or two episodes of conductive hearing loss during the study.

**Table 8:** Number of children according to episodes of conductive hearing loss in each

Group	Children with one episode of otitis	Chidlren with two episodes of otitis	Chidlren that did not present otitis	Total
Control group	22(12%)	1 (0,54%)	160 (87,4%)	183(100%)
Study group	7 (20,6%)	3 (8,8%)	24(70,5%)	34(100%)
Total	29	4	184	217

Key: n = number

Follow-up of newborn subjects across 12 months after hearing screening revealed that most of the children presented at least one episode of otitis.

Tables 9 and 10 show the occurrence of conductive hearing loss according to the variable sex.

**Table 9:** Occurrence of conductive hearing loss according to sex in the control group

			Number of children with conductive loss	
Sex	n		%	P-Value
Female	10		43,4	0,8329
Male	13		56,5	
otal	23		100	

Key: n=number

**Table 10:** Occurrence of conductive hearing loss according to sex in the control group

			Number of children with conductive loss	
Sex	n		%	P-Value
Female	4		43,5	0,3754
Male	6		60,0	
Total	10		100	

Key: n=number

Although males had a higher occurrence of conductive hearing loss, this difference was not statistically significant (Tables 9 and 10).

## DISCUSSION

Among the middle ear changes in childhood episodes of recurring and/or persistent otitis and temporary hearing losses have come to the attention of healthcare professionals.

The occurrence of conductive hearing loss due to secretory otitis media in children that failed in hearing screening (conductive hearing loss) while monitored during 12 months was analyzed.

The results were similar in both studies; the discussion, therefore, will be done jointly.

Analysis of the occurrence of conductive hearing loss in both groups revealed that there was a statistically significant difference between the study group and the control group; this result was common to the prospective and retrospective studies (Tables 1 and 6).

We found in the prospective study that 16 children (57.1%) in the study group presented conductive hearing loss within the first year of life; in the control group this number was nine (23.7%). These results are similar to those of Doyle et al. (2004);6 these authors studied whether neonates with persistent middle ear effusion at 30 to 48 hours of life had or not an increased tendency to present otitis media within the first year of life compared to neonates with no effusion. They found that eight children (58%) of 14 in the study group presented otitis media in the first year of life; three (20%) of 15 children in the control group presented otitis media in the first year of life. This difference was statistically significant, as was the case in the present study.

Although there was a higher rate of conductive hearing loss in the study group, children in the control group also presented conductive hearing loss within the first year of life: 23.7% in the prospective study, and 12.6% in the retrospective study (Tables 1 and 6).

Conductive hearing loss may be a complication of otitis media, especially secretory otitis media. This condition is one of the most common diseases in childhood (Sih et al., 1993).^12^ Sutton et al. (1996)^13^ have stated that middle ear effusion may occur in 50% of the ears of neonates, which may result in hearing screening failure (done with otoacoustic emissions). Klein (1989)^14^ studied 2,500 children aged below 2 years and found that 42% of them had occasional otitis media, and 33% had recurring otitis media (over three episodes). Hogan et al. (1997)^15^ carried out a longitudinal study of 112 neonates that were monitored for three years, and found that children aged below 2 years are more susceptible to recurring episodes of effusion, concluding that for this reason special care should be given to neonates. Isaac et al. (1999)^16^ reported that otitis media starts within the first months of life in some children and becomes recurrent; many of these children continue with recurring episodes of otitis media and chronic otitis media with fluctuation of their hearing during important years for language and speech development. Our results support Hogan et al.'s (1997)^15^ and Isaac et al.'s (1999) findings.^16^

Observations in the present study showed that neonates that had otitis in the neonatal period (1st month of life) had a higher possibility of presenting other episodes of otitis within 12 months. This finding is similar to Saes et al.'s (2005)^4^ results on the occurrence of otitis (middle ear effusion) in 190 neonates, showing that the moment of the first episode of otitis correlated directly with its recurrence. Children who presented a first episode of otitis after 6 months of age had a lower recurrence rate. The opposite was seen in cases where the first episode of otitis occurred within 6 months of life; in these cases, the recurrence rate was higher. The study group in our study – children that had a first episode of otitis within the first month of life – had a higher number of recurrences compared to children that did not present otitis within the first month of life (controls) (Tables 1 and 6).

Paparella (1972)^17^ showed that spontaneous improvement could occur without losses in language acquisition or with no other sequels in some cases of otitis media. In other cases, the disease may become chronic and lead to learning impairment and language difficulties. Furthermore, if untreated, the disease may affect the mastoid cells or even the cranial cavity, resulting in severe complications; it may also reach the inner ear and cause sensorineural hearing loss.

Although males had a higher rate of conductive hearing loss, this difference was not statistically significant (Tables 4, 5, 9 and 10). Sih (1998)^18^ showed that male children had a higher possibility of presenting otitis media. Saes et al. (2005)^4^ studied the occurrence of episodes of otitis (middle ear effusion) in 190 neonates. These authors found a higher frequency (four to six episodes of otitis) in male infants, which was statistically significant; this topic remains controversial. Most researchers have reported that there are no gender differences in the incidence of otitis media with effusion or in duration of middle ear effusion (Paradise et al, 1997).^19^ A few studies have shown that boys have a significantly higher incidence of acute otitis media and more episodes of acute otitis media than girls (Teele et al, 1989);^20^ other authors, however, have not confirmed these findings (Casselbrant et al, 1995).^21^ Descriptions have shown that boys have a higher propensity for presenting persistent middle ear effusion. The reason for this gender difference remains unknown.

Studies have been made to find in which of the assessments (2nd assessment at six months and a 3rd assessment at 12 months) the rate of conductive hearing loss was higher among the children with conductive hearing loss within 12 months of life. The peak occurrence of conductive hearing loss in the study group (in both studies) was age six months (retrospective – 53.8%; prospective – 55%) (Tables 2 and 7). This finding corroborates Saes et al.'s (2005)^4^ findings, which showed that the onset of the first episode was directly associated with recurrence.

Engel et al. (1999)^22^ undertook a prospective longitudinal study to verify the prevalence of otitis media with effusion in 150 healthy neonates and 100 high risk neonates aged from zero to two years. As in the present study, there were differences in both groups (high risk and normal infants) that remained at least until 24 months as children developed. The peak incidence of otitis media with effusion (59% in the high risk group versus 49% in the normal group) was observed around age ten months. These authors concluded that the prevalence of otitis media with effusion is high during infancy, especially in high risk children.

Saes et al. (2005)^4^ studied the occurrence of otitis in 190 neonates and concluded that the highest incidence of middle ear effusion was during the first year of life, peaking between ages four and 12 months. Fiellau-Nikolajsen (1990)^5^ reported that secretory otitis media is relatively uncommon in the first six months of life. The incidence increases markedly between ages six and 12 months, reaching 13 to 15% at age 12 months. These results are similar to our findings (otitis peaking at age 6 months). Costa et al. (1994)^23^ reported that the incidence of secretory otitis media is higher at ages six to eight months, and from 4 to 6 years; the trend decreases with age. Roy et al. (2007)^24^ conducted a retrospective study to investigate the incidence of otitis media within the first two years of life in a population of 252 neonates in Bangladesh. These authors found the highest occurrence of secretory otitis media from ages 6 and 12 months, and the lowest occurrence within the first three months of life.

Monitoring of neonates in 12 months after hearing screening showed that there was only one episode or conductive hearing loss (due to otitis) per child after the second assessment in the prospective and retrospective studies.

There was a higher occurrence of only one episode of conductive loss in both studies (Tables 3 and 8). The one-year occurrence in this study is similar to that in a study by Roy et al. (2007).^24^ These authors investigated the incidence of otitis media within the first two years of life in a population of 252 neonates and found an incidence rate of 0.9 episodes per child-year. Among these patients, 46% (n = 115) of 252 subjects presented otitis, of which 36% (n = 91) within the first year of life, and 10% (n = 24) during the second year of life (p <0.001). Klein (1989)^14^ studied 2,500 children aged less than two years and found that 42% presented otitis media occasionally, and 33% presented recurring otitis media (over three episodes a year).

These results show that identifying conductive hearing loss in neonatal and infant hearing screening is an important goal so that prompt and appropriate interventions may be undertaken; although it may be transitory, conductive hearing loss may result in altered language acquisition and auditory processing. Several studies in the literature we reviewed have underlined the importance of the diagnosis of conductive losses (Northern, Downs, 1991^2^; Klein, 1989^14^; Hogan et al., 1997^15^; Zapala, 1997^25^; Doyle et al., 199726; Engel et al., 1999^22^; Cone-Wesson et al., 1997^27^; Doyle et al., 2004^6^; Saes et al., 20 05^4^; Roy et al., 2007^24^) and have noted the elevated incidence of this condition in children at a higher risk.

A higher occurrence of otitis in children that fail in neonatal hearing points at the need for joint work with otorhinolaryngologists. Thus, it is essential for such a professional to be part of the neonatal hearing screening team and to participate in audiological monitoring programs of children.

## CONCLUSION

Analysis of our results showed that neonates who failed in hearing screening done in the first months of life- because of secretory otitis media – have a higher chance of developing episodes of otitis, especially between 6 and 12 months of life. A second conclusion is that neonatal hearing screening programs require measures by otorhinolaryngologists to diagnose auditory changes, especially in the middle ear, which may result in future auditory processing losses, with resulting learning disabilities.
